# The Emerging Role of Neutrophil Extracellular Traps (NETs) in Tumor Progression and Metastasis

**DOI:** 10.3389/fimmu.2020.01749

**Published:** 2020-09-16

**Authors:** Maria Teresa Masucci, Michele Minopoli, Silvana Del Vecchio, Maria Vincenza Carriero

**Affiliations:** ^1^Neoplastic Progression Unit, Istituto Nazionale Tumori IRCCS “Fondazione G. Pascale”, Naples, Italy; ^2^Department of Advanced Biomedical Sciences, University of Naples “Federico II”, Naples, Italy

**Keywords:** NETosis, tumor associated neutrophils (TANs), neutrophil extracellular trap (NET), tumor microenvironment (TEM), tumors

## Abstract

Neutrophil Extracellular Traps (NETs) are net-like structures composed of DNA-histone complexes and proteins released by activated neutrophils. In addition to their key role in the neutrophil innate immune response, NETs are also involved in autoimmune diseases, like systemic lupus erythematosus, rheumatoid arthritis, psoriasis, and in other non-infectious pathological processes, as coagulation disorders, thrombosis, diabetes, atherosclerosis, vasculitis, and cancer. Recently, a large body of evidence indicates that NETs are involved in cancer progression and metastatic dissemination, both in animal models and cancer patients. Interestingly, a close correlation between cancer cell recruitment of neutrophils in the tumor microenvironment (Tumor Associated Neutrophils. TANs) and NET formation has been also observed either in primary tumors and metastatic sites. Moreover, NETs can also catch circulating cancer cells and promote metastasis. Furthermore, it has been reported that wake dormant cancer cells, causing tumor relapse and metastasis. This review will primarily focus on the pro-tumorigenic activity of NETs in tumors highlighting their ability to serve as a potential target for cancer therapy.

## Introduction

Neutrophil Extracellular Traps (NETs) are web-like structures, discovered by Volker Brinkmann and Arturo Zychlinsky in 2004, able to entrap bacteria fungi, protozoa, and virus (1). They are extruded by activated neutrophils and are composed of DNA fibers, histones, and antimicrobial proteins (2–4), in which pathogens are immobilized and exposed to a local high and lethal concentration of effector proteins (5). Zychlinsky's group has demonstrated that NETs kill bacteria, thus defining a new modality of antimicrobial innate response (1).

Besides their role as a host defense mechanism, NETs play a pivotal role in non-infectious conditions, such as systemic lupus erythematosus (6), rheumatoid arthritis, cystic fibrosis (7, 8), and psoriasis (9). They are also involved in other pathological processes, such as coagulation disorders, thrombosis (10), diabetes (11, 12), atherosclerosis (13), vasculitis (14), wound healing (12), and periodontitis (15, 16). Recently, several studies have investigated the role of NETs in tumors and reported their involvement in cancer immunoediting (3), progression (3, 17–19), metastatic spread (20), and cancer associated thrombosis (20). NETs can exert an anti-tumor or pro-tumor activity. NET components like myeloperoxidase (MPO) (21), proteinases (20, 22), and histones (23) can kill a tumor, inhibit tumor growth, and metastasis (21). NET proteinases can also degrade the extracellular matrix, promoting cancer cell extravasation, and metastasis (20, 22).

Furthermore, previous studies showed the ability of NETs to entrap and serve as an adhesion substrate for cancer cells (24, 25) thus promoting metastatic dissemination. NETs have been identified in peripheral blood and tumor specimens from animal models and cancer patients (26), but their role has not been completely elucidated. Moreover, circulating NETs are supposed to cause organ damage in cancer patients, similar to those occurring in auto-immune diseases (27), and their ability to awake dormant cancer cells has also been reported (28).

This review will primarily focus on the pro-tumorigenic activity of NETs in tumors, highlighting their ability to serve as a potential target for cancer therapy.

## NETosis

The process by which neutrophils extrude NETs is known as NETosis. Different signals and mechanisms are involved in NET formation (29–31). Grayson and Kaplan suggested that the production of Reactive Oxygen Species (ROS), migration to the nucleus of neutrophil elastase (NE) and MPO, histone modification, and de-condensation are the pivotal events of NETosis (29). Sørensen and Borregaard proposed that NET formation pathways *in vitro* and *in vivo* could be distinct since the citrullination of histones by peptidyl arginine deiminase 4 (PAD4) and the consequent unwrapping of nucleosome are central events of NET formation *in vivo* (32, 33).

NETosis is considered a type of cell death, different from apoptosis and necrosis characterized by the release of de-condensed chromatin and granular contents to the extracellular space (34). NETosis usually needs the stimulation of neutrophils and the generation of ROS by NADPH oxidase (29, 35, 36). NETs generated from mitochondrial DNA are described (37) being the amount of mtDNA 100,000 times smaller than nuclear DNA because mitochondria are very few in neutrophils, but whether this difference plays any role, needs to be investigated further (38).

In addition to infectious and non-infectious diseases, NETs formation has been described also in the tumor microenvironment (39, 40). Cancer cell granulocyte-colony-stimulating factor (G-CSF) and endothelial IL-8 are considered the main triggers of NETosis in tumors (41, 42). The over-expression of G-CSF in cancer causes a higher number of neutrophils in the blood, ROS generation, and subsequent NETosis (43, 44). Gupta et al. demonstrated that activated Endothelial Cells (ECs), co-cultured with neutrophils, can promote the NET formation, partially mediated by IL-8 (42). Finally, pancreatic cancer (PaCa) cells were shown to stimulate NETosis, directly or via PaCa-primed platelets (45).

## Presence and Role of Nets in Tumors

NETs have been identified in the blood and tumors of both animal models and patients where they may serve as pro- or anti-tumoral factors, depending on the status of the immune system or tumor microenvironment (20, 31). In particular, the correlation between NET formation, tumorigenesis, tumor progression, and metastasis has been investigated and there have been reports of the direct activity of NETs on tumor cell proliferation, through their proteases or activating signals (3, 19, 20, 46). Furthermore, the ability of cancer cells to promote NETosis *in vivo* and *in vitro* (41) and the presence of Tumor Associated Neutrophils (TANs) in the tumor microenvironment, as well as their association with NET formation, have been demonstrated (41, 47–49).

In the next two sections, we provide an overview of studies on the role of NETs in tumor models and cancer patients.

### NETs in Tumor Models

Several studies showed that cancer cells can induce the release of NETs by neutrophils. In chronic myelogenous leukemia, mammary, and lung carcinoma mouse models, circulating neutrophils were more prone to release NETs than those of healthy animals. The high tendency of neutrophils to form NETs in these animals correlated to cancer systemic effects on the host (43, 50).

In two transgenic mouse tumor models, RIP1-Tag2 of insulinoma and MMTV-PyMT of breast cancer, vascular alterations, and enhanced neutrophil infiltration in the heart and kidneys, resembling tumor-induced systemic damage of cancer patients, were observed. Moreover, platelet–neutrophil complexes, indicative of NETs formation were found in the kidneys. DNase treatment completely restored kidney vascular function to levels seen in healthy mice, suggesting the causal role of NETs in the impaired vascular function of tumor-bearing mice (27). In pancreatic adenocarcinoma (PDA), NETs and platelets have been demonstrated to play a pivotal role in blood hypercoagulability, resulting in an increased risk of venous thromboembolism, and in cancer-associated thrombosis, both in an orthotopic PDA model in C57/Bl6 mice and patients (51).

An *in vitro* study showed that a pancreatic cancer cell line (AsPC-1) induced the formation of NETs in a dose-dependent manner (17) and the major role in NETosis was attributed to protein components of AsPC-1 conditioned medium (CM). In the same study, NETs were shown to enhance the endogenous thrombin potential (ETP) of normal plasma and induce cancer cell migration, invasion, and angiogenesis (17). In another *in vitro* study, it was demonstrated that Lewis Lung Carcinoma (LLC) extracellular RNAs (exRNA) cause the formation of NETs (52).

Furthermore, spontaneous intestinal tumorigenesis correlates with the accumulation of low-density neutrophils with a pro-tumorigenic N2 phenotype, upregulation of complement C3a receptor, and NET formation (53).

The role of NETs in promoting tumor metastasis seems to be very important, and it has been reported that NETs can entrap circulating tumor cells (CTCs) thus favoring the metastatic dissemination. In a tumor murine model, mimicking the post-operative inflammatory environment, adhesion of cancer cells to NETs was demonstrated, β1-integrin expression was upregulated and the expression of β1-integrin on both cancer cells and NETs was essential for the adhesion of CTCs to NETs, *in vitro*, and *in vivo*. DNase treatment abrogated cancer cell adhesion to NETs (54). In a mouse model, NET formation and entrapment of circulating lung tumor cells caused a higher number of hepatic micro-metastasis (55). Finally, NETs were able to promote the development and progression of liver metastases after surgical stress (56).

**Figure 1:**
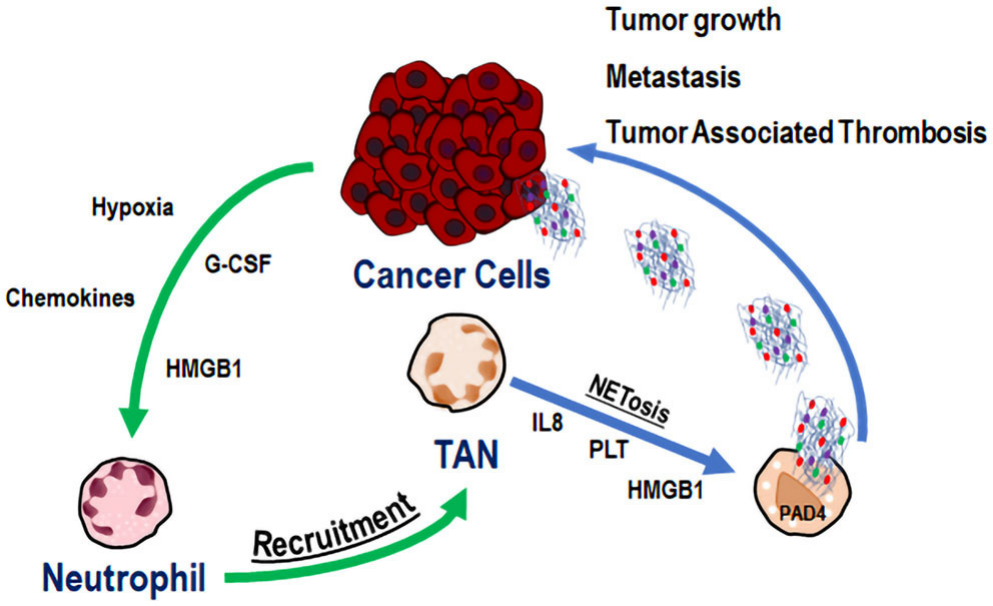
Cancer cell, TAN, and NET co-operation in tumor progression and metastasis. Cancer cells recruit neutrophils to tumor microenvironment through several signals (green arrows). In tumor microenvironment Tumor Associated Neutrophils (TANs) are activated to release Neutrophil Extracellular Traps (NETs) (blue arrows), able to promote tumor growth, tumor progression, metastasis, and tumor associated thrombosis.

Another issue that has been addressed is the close interaction between cancer cells, TANs, and NETs (Figure 1). Recently, Tohme et al. showed that NETs promoted tumor cell growth by enhancing their mitochondrial functions. Moreover, subcutaneous implanted tumors grew faster in WT than in PAD4-KO mice and liver metastases were fewer in PAD4-KO compared to WT mice. Similar results were obtained by intra-peritoneal administration of DNase to WT mice. The immunofluorescent staining of tumor tissues from PAD4-KO tumors showed a very low neutrophil infiltration as compared to that of WT mice. Neutrophil recruitment by conditioned media from hypoxic cancer cells was observed *in vitro* and was mediated by high levels of chemokines and HMGB1, also able to generate NETs within the tumor microenvironment. Altogether, these data highlight the centrality of neutrophil recruitment and NET formation for tumor growth and progression (49).

In another interesting study, Park et al. highlighted the close relationship between metastatic cancer cells, neutrophil recruitment, and NET formation. They showed that metastatic breast cancer cells stimulate neutrophils to form metastasis-supporting NETs. CXCL1 was able to mediate neutrophil recruitment into tumors from mice orthotopically transplanted with 4T1 (metastatic) and 4T07 (non-metastatic) murine breast cancer cells. More neutrophils were found in primary 4T1 tumors than in 4T07 tumors and the reduction of CXCL1 in 4T1 cells lowered neutrophil infiltration into tumor lesions. By immuno-fluorescent staining for NETs of lung tissue sections from mice specimens, the NET formation was shown to occur just after 4T1 cell tail vein injection. Moreover, G-CSF makes metastatic cancer cells form NETs at sites of dissemination; and anti-G-CSF blocking antibodies highly reduce the ability of 4T1 cells to induce NETosis, in a trans-well chamber assay (41).

Besides all the previously described functions, NETs “wake-up” dormant tumor cells and cause metastasis. Recently, Albrengues et al. (28) demonstrated a clear role of NETs in tumor relapsing. In mice, the mechanism by which the dormant cells wake-up and produce metastases was demonstrated. Chronic lung inflammation caused by tobacco smoke or nasal instillation of LPS promoted lung metastases, due to the formation of NETs and suppressed *in vitro* and *in vivo* by digesting NET-DNA with DNase. Moreover, NETs bind the extracellular matrix and provoke laminin cleavage and remodeling, with the exposition of a new epitope that awakes the dormant cell proliferation, through integrin activation and FAK/ERK/MLCK/YAP signaling.

Different techniques could be used for the detection of adhesive interactions between NETs and tumor cells. Monti et al. (24) demonstrated that various cancer cell lines (HT1080, U-87 MG, H1975, DU 145, PC-3, and A-431) were able to adhere *in vitro* to neutrophil-like cells derived NETs, via α5β1, αvβ3, and αvβ5 integrins expressed on the cell surface. An excess of cyclic RGD peptide inhibited cancer cell adhesion to NETs, at a rate similar to that obtained with DNase treatment. This approach can be used to screen cancer cells expressing RGD-binding integrins for their adhesion capability to NETs.

### NETs in Cancer Patients

NETs have been correlated to cancer progression and metastatic spread also in human tumors. Levels of NETs in plasma of patients with different types of tumors including lung, pancreatic, and bladder cancer, were found to be higher than in healthy controls (57). In lung cancer patients, Li et al. demonstrated the presence of NETs in lung tissues, peripheral blood, and sputum (52). Moreover, in colorectal cancer patients, the levels of NETs produced by neutrophils after *in vitro* stimulation were significantly higher than in the healthy controls and associated with a poor clinical outcome (58).

Park et al. demonstrated the presence of NETs in human breast cancer by immuno-fluorescence staining. NETs were detected in metastatic lung lesions with the highest percentage of positivity in triple-negative tumors (41). Similarly, in histopathological samples of colorectal liver metastases from patients who underwent elective curative resection, Tohme et al. found a higher presence of TANs and NETs compared to normal liver. Also, tumor tissue expressed high levels of citrullinated histones, indicative of NETosis. Furthermore, preoperative levels of MPO-DNA, a reliable marker for systemic NETosis (56), were higher in patient serum than in healthy controls and were associated with poor DFS and OS. Thus, MPO-DNA serum levels could represent a possible prognostic biomarker in these patients (49).

The relationship between TANs and NET formation in patient tumors and its role in tumor immuno-editing has been investigated particularly in pediatric Ewing sarcoma. Berger-Achituv et al. demonstrated the presence of TANs in diagnostic biopsies of Ewing Sarcoma pediatric patients. In two of these, NETs are derived from TANs activation. These two patients presented metastases and early relapse after high doses of chemotherapy, suggesting a possible role of NETs in Ewing Sarcoma progression (3).

The importance of NETs in cancer has been also highlighted by the relation of NETs with alteration of coagulation in tumor patients. It has been shown that NETs stimulate cancer-associated thrombosis in tumors, a phenomenon associated with a very bad prognosis in patients (18, 19). The circulating levels of NETs were measured in HCC patients, by evaluating their markers (DNA-histone complex, double-stranded DNA, neutrophil elastase). Similarly, contact system activation markers were measured. NETs and contact system activation markers were higher in HCC patients than in healthy controls (59). Jung et al. measured the circulating levels of NETs and hypercoagulability markers in patients with pancreatobiliary malignancies and found a correlation between high levels of NET markers and hypercoagulability markers (17). Moreover, higher levels of plasma Citrullinated histone H3 (H3Cit) were observed in cancer patients in advanced stages, compared to those of a healthy individual, and neutrophils from cancer patients showed a higher content of H3Cit than others. Furthermore, H3Cit in plasma of cancer patients does correlate with NE, MPO, IL-6, and IL-8, all activators of NETosis (56, 60).

## NETs as Potential Targets in Cancer Therapy

**Figure 2:**
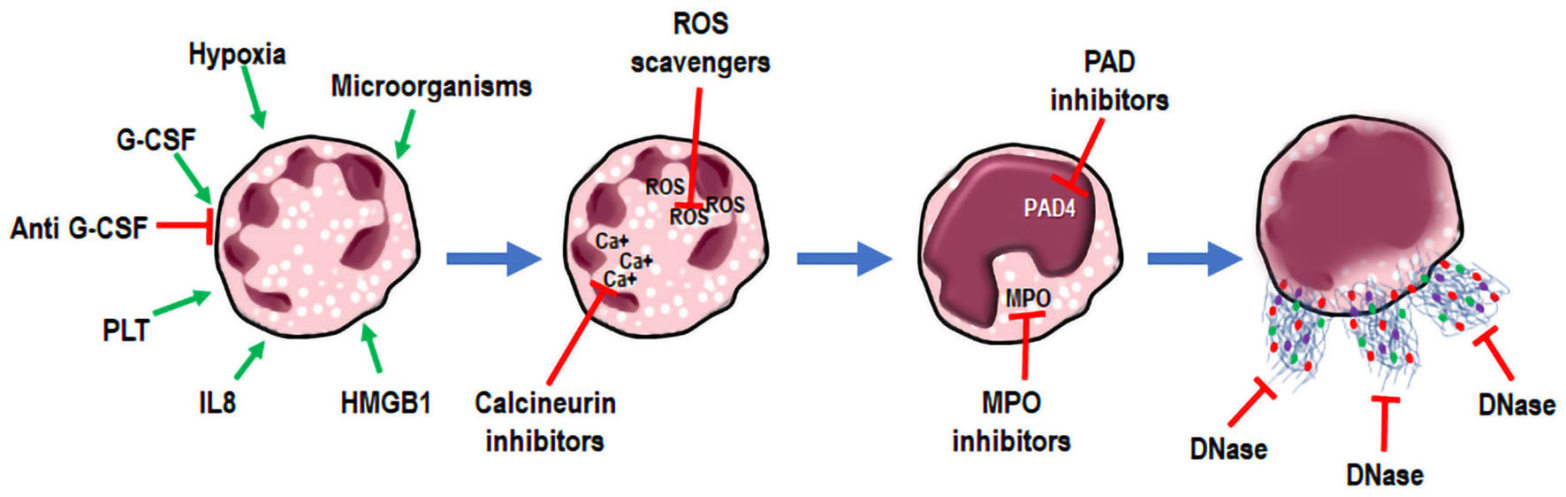
NETosis. NETs as targets for therapy. Neutrophil stimulation (green arrows) results in NETosis and extrusion of Neutrophil Extracellular Traps (NETs) (blue arrows). During these processes, targets for therapies have been postulated, and interfering drugs (red arrows) have been already used in clinical practice or are under investigation *in vivo*.

NETs can be regarded as promising therapeutic targets in cancer (Figure 2). Taking into account the role of NETs in enhancing the metastatic potential of cancer cells, it could be advantageous to inhibit NET formation and/or activity in tumors (41). Unfortunately, the ongoing clinical trials have not yet clarified the best modality to target NETs (31, 61). Gonzalez-Aparicio et al. have suggested the combination of immunotherapy strategies and drugs interfering with neutrophil chemoattraction and NET extrusion, as a possible treatment of cancer (39). Another issue to be considered is the actual lack of markers that can predict a good response to NETs interfering therapy in patients. The only available markers of NET formation are H3Cit and MPO-DNA, which may have prognostic value in cancer patients (56, 60). The detection of NETs in tumor biopsies and the expression of G-CSF in human tumor samples were proposed as patient selection criteria for NET targeting therapies, but these studies did not provide conclusive evidence (41, 62). The semi-quantitative method for the identification of NETs in body fluids, based on the association of DNA with MPO, needs more investigation (63). The majority of experimental and clinical studies targeting NETs have been accomplished in pathologies other than cancer, such as autoimmune and pulmonary diseases or complications of autoimmune conditions, as reviewed by Jorch and Kubes (64).

In Systemic lupus erythematosus (SLE), it has been demonstrated that the serum endonuclease DNase is essential to degrade chromatin within NETs. Moreover, in some SLE patients, DNase dis-functions cause very severe kidney injuries, underlining the importance of a good balance in NET formation and degradation (65). Therefore, considering that the DNase can disrupt NET structure, NETs could represent a good target for DNase therapy (65). Based on this evidence, DNase has been tested in experimental cancer models. For instance, DNase treatment decreased disease burden in breast cancer mouse models (27). Moreover, in a mouse model of intra-abdominal sepsis mimicking cancer post-operative inflammatory environment, NETs trapping of circulating tumor cells and adhesion was abrogated, *in vivo*, by DNase (54). The systemic administration of DNase was also shown to slightly reduce experimental metastasis in a mouse model of metastatic lung cancer (55). On the contrary, DNase coated nanoparticles exerted a higher effect, due to the stabilization of DNase linked to nanoparticles in blood. DNase nanoparticles digested NETs *in vitro* and block breast cancer lung metastasis formation, *in vivo*, whereas they did not affect primary tumor growth (41, 62). These results in animal models stimulate the use of DNase in human cancer, with no toxicity in clinical studies. DNase treatment was approved by the FDA for cystic fibrosis patients, suffering from excessive NET release in their lungs (66) to dissolve dense mucus (4). Inhibitors of molecules involved in NETosis, and interfering with NET formation, used in non-cancer pathologies, could be tested in cancer patients. Among these, NE inhibitors, used in Chronic Obstructive Pulmonary Disease and inhibitors of PDA4 could improve the clinical outcome of cancer patients (4), even if the commercially available PAD4 inhibitors Cl-amidine have short serum half-lives (67). Domingo-Gonzalez et al. demonstrated that PGE_2_ via EP2 or EP4, negatively regulates NETosis, both in mice and in patients transplanted with hematopoietic stem cells, thus revealing another physiologic substance able to antagonize NET formation (68). In another study (17), PGE1 was shown to inhibit both cancer cell- and PMA- induced NET formation, probably by the induction of intracellular cAMP and the reduction of intracellular calcium ions, necessary to form NETs (69). In the same study, anti-thrombin significantly reduced NET formation induced by cancer cells (17). Furthermore, the NET inhibitor chloroquine reduced blood platelet aggregation, circulating tissue factor, and decreasing hypercoagulability in tumor bearing mice. Interestingly, the same effects were identified in patients (51). In a recent review, Hector Salazar-Gonzalez and colleagues examined the role of NETs in renal diseases, highlighting the necessity to block NET formation to interfere with the pathogenesis of inflammatory and non-inflammatory diseases, including cancer (70). Finally, beyond the role of NETs as a possible therapeutic target in cancer, a direct therapeutic role of NETs has been reported in bladder cancer. NETs induced by Bacillus Calmette-Guerin (BCG) in the early stage of immunotherapy play an immune-regulatory role, by recruiting T cells and monocytes-macrophages, thus preventing tumor growth. Moreover, BCG induced NETs cause apoptosis and cell-cycle arrest, and inhibit cancer cell migration (71). Recently, the same group demonstrated that an immune reaction involving neutrophils is caused by BCG intravescical BCG infusion in the early stages of the treatment. BCG induces NET release in a time dependent manner, requiring ROS, being MEK, p38, PI3K, and PKC necessary to regulate the all process.

## Conclusions and Future Directions

The close cooperation between cancer cells, TANs, and NET formation in the tumor microenvironment highlights the function of NETs in cancer progression and metastasis. NETs are able to wake up dormant cancer cells, promoting cancer relapse and metastasis. In addition, they are also able to entrap circulating cancer cells and enhance metastasis spread.

Further studies are needed to investigate the potential role of NETs as targets for cancer therapy and to evaluate the possibility of pharmacologically interfering with NET formation and destruction, as it has been accomplished in autoimmune diseases. Promising results were obtained in tumor models whereas in cancer patients recent studies did not provide conclusive evidence of the efficacy of this approach. Indeed, it will be necessary to focus future research on investigating the regulation of NETosis and the balance between NET formation and destruction, in order to pharmacologically target NETs, without hampering immune functions. Thereafter, clinical trials testing the efficacy of NETs interfering drugs in cancer patients could be designed.

## Author Contributions

MTM and MC: conception of the work. MTM and MM: extensive literature search and manuscript drafting. MTM, SD, and MC: critical revision of the work and final version approval. All authors contributed to the article and approved the submitted version.

## Conflict of Interest

The authors declare that the research was conducted in the absence of any commercial or financial relationships that could be construed as a potential conflict of interest.
